# Cryptococcal Meningitis: Differences between Patients with and without HIV-Infection

**DOI:** 10.3390/pathogens12030427

**Published:** 2023-03-08

**Authors:** Chutithep Teekaput, Saowaluck Yasri, Romanee Chaiwarith

**Affiliations:** 1 Division of Neurology, Department of Internal Medicine, Faculty of Medicine Chiang Mai University, Chiang Mai 50200, Thailand; 2 Division of Infectious Diseases and Tropical Medicine, Department of Internal Medicine, Faculty of Medicine Chiang Mai University, Chiang Mai 50200, Thailand

**Keywords:** cryptococcal meningitis, meningitis, invasive fungal infection, HIV-infection

## Abstract

**Background:** Cryptococcal meningitis is one of the most devastating infections, particularly in HIV-infected individuals. The increased use of immunosuppressants led to an increase in the incidence of cryptococcosis in HIV-uninfected individuals. This study aimed to compare the characteristics between groups. **Methods:** This retrospective cohort study was conducted from 2011 to 2021 in northern Thailand. Individuals diagnosed with cryptococcal meningitis aged ≥15 years were enrolled onto the study. **Results**: Out of 147 patients, 101 were individuals infected with HIV and 46 were non-infected. Factors associated with being infected with HIV included age < 45 years (OR 8.70, 95% CI 1.78–42.62), white blood cells < 5000 cells/cu.mm. (OR 7.18, 95% CI 1.45–35.61), and presence of fungemia (OR 5.86, 95% CI 1.17–42.62). Overall, the mortality rate was 24% (18% in HIV-infected vs. 37% in HIV-uninfected individuals, *p*-value = 0.020). Factors associated with mortality included concurrent pneumocystis pneumonia (HR 5.44, 95% CI 1.55–19.15), presence of alteration of consciousness (HR 2.94, 95% CI 1.42–6.10), infection caused by members of *C. gattii* species complex (HR 4.19, 95% CI 1.39–12.62), and anemia (HR 3.17, 95% CI 1.17–8.59). **Conclusions:** Clinical manifestations of cryptococcal meningitis differed between patients with and without HIV-infection in some aspects. Increasing awareness in physicians of this disease in HIV-uninfected individuals may prompt earlier diagnosis and timely treatment.

## 1. Introduction

Cryptococcal meningitis is a devastating neuroinfectious disease caused by members of the *Cryptococcus neoformans***/***C. gattii* species complex (CNGSC) [1]. Infection with human immunodeficiency virus (HIV) was the main underlying condition associated with this disease [2]. In 2009 the global burden of cryptococcal meningitis published was an estimated 957,900 cases of cryptococcal meningitis annually among HIV patients, resulting in 624,700 fatalities within three months of infection. Although most cases occurred in sub-Saharan Africa, 120,000 cases each year were reported in Southeast Asia with a three-month mortality rate of 55% [2]. A Thai study reported that cryptococcal meningitis was the third most common opportunistic infection after tuberculosis and pneumocystis pneumonia; however, it is the most common infection affecting the central nervous system in HIV patients [3]. During the past two decades, the prevalence of cryptococcal meningitis in HIV-uninfected patients increased due to the widespread use of immunosuppressive therapy and the increasing number of recipients receiving transplants [4,5,6,7,8]. For example, the 14-year US-based study demonstrated that in the first seven years of the study, half of the patients were HIV-infected individuals and the number dropped to less than one-third in the following seven years [8]. Several studies of cryptococcal meningitis in patients with adult-onset immunodeficiency syndrome (AOID) caused by anti- interferon-γ autoantibody (anti-IFN-γ AAb) were reported [9,10]. Comparisons of clinical characteristics of cryptococcal meningitis between patients with and without HIV-infection were reported infrequently in Thailand, e.g., 2004 and 2008. In this study, we describe clinical features and outcomes of cryptococcal meningitis between patients with and without HIV-infection in northern Thailand.

## 2. Materials and Methods

A retrospective cohort study was conducted between 1 January 2011 and 31 December 2021 at Maharaj Nakorn Chiang Mai Hospital, a 1400-bed, tertiary-care, university-affiliated hospital in northern Thailand. Participants were recruited if they were ≥15 years of age and had a laboratory-confirmed diagnosis of cryptococcal meningitis detected by one of the following: (1) presence of encapsulated budding yeast cells from the India ink preparation, (2) presence of encapsulated budding yeast cells from histological investigation of clinical specimens, (3) presence of cryptococcal polysaccharide antigens in the cerebrospinal fluid, or (4) culture growth of *Cryptococcus* species. Data extracted from the medical records included demographic and clinical characteristics, laboratory findings, details of therapy, and treatment outcomes, i.e., overall mortality at discharge, 30day, 90day, and 1 year time periods.

### Statistical Analysis

Clinical data are presented as numbers (%), and mean and standard deviation (SD), or median and interquartile range (IQR) as appropriate. Comparisons between groups were analyzed using Student’s t-test, Mann–Whitney U test, chi-square test or Fisher’s exact test as appropriate. Multivariate logistic regression models were used to identify factors associated with underlying HIV-serostatus. Time to death comparing between groups were analyzed using Kaplan–Meier estimates and log rank tests. Patients who survived were censored at 1 year after diagnosis of cryptococcal meningitis. Cox-proportional hazard models were also used to identify factors that were predictive of death. Variables with a *p*-value of < 0.10 from the univariable models were then tested in multivariable models. A two-sided test at a *p*-value of < 0.05 was used to indicate statistical significance. All statistical analyses were performed using Stata statistical software version 16.0 (Stata Statistical Software, Texus, USA: Release 16.0, Stata Corporation, College Station, TX, 2019).

## 3. Results

### 3.1. Demographic Clinical Characteristics

**Figure 1:**
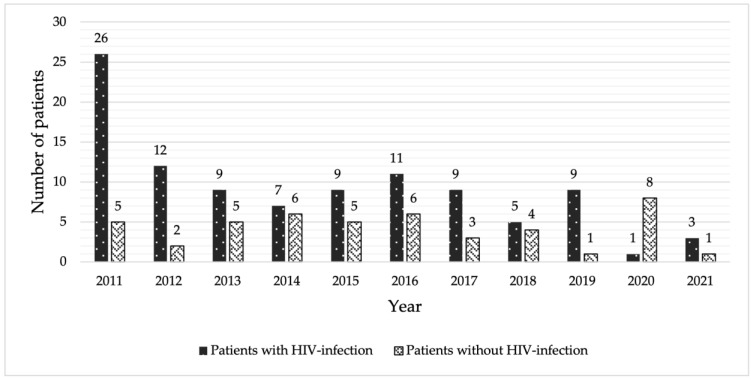
Number of patients with cryptococcal meningitis by years of diagnosis and HIV-serostatus.

During the 10-year period, 152 patients met the inclusion criteria and were included in the study. Five patients had incomplete data and were excluded. Out of the remaining 147 patients, 101 (68.7%) and 46 (31.3%) were patients with and without HIV-infection, respectively. The number of cases by year and HIV-serostatus is shown in Figure 1. Sixty-six (65.4%) and 29 (63.0%) patients with and without HIV-infection were male, and the mean age was 45.0 ± 12.4 and 61.9 ± 16.3 years, respectively. In the HIV-infected patients, the most common route of HIV transmission was heterosexual (64 patients, 63.4%), followed by homosexual (25 patients, 24.8%) and intravenous drug user (12 patients, 11.9%). The median (IQR) CD4 cell counts from 96 patients were 23 cells/cu.mm. (10, 68), with 83 out of 96 patients (86.5%), 9 (9.4%), and 4 (4.2%) having CD4 < 100, 100–200, and > 200 cells/cu.mm, respectively. Cryptococcal meningitis was the first presentation at HIV diagnosis in 59 patients (58.4%). The two common concurrent opportunistic infections (OIs) were oral or esophageal candidiasis and tuberculosis.

Out of the 46 HIV-uninfected individuals, 12 patients (26.1%) received B-cell immunosuppressive drugs along with steroid therapy, 5 patients (10.9%) had cancer and received targeted therapy, 3 patients (6.5%) had AOID caused by anti-IFN-γ AAb, and 1 patient (2.2%) was a solid organ transplant recipient. No underlying disease was identified in the remaining 25 patients. 

In comparison with HIV-uninfected individuals, patients with HIV-infection were more likely to be younger, have a higher Charlson comorbidity index (CCI), have a higher incidence of nausea/vomiting, and were less likely to experience an alteration of consciousness (Table 1).

### 3.2. Laboratory Findings

In comparison with HIV-uninfected individuals, patients with HIV-infection were more anemic, had lower amounts of white blood cells, lower platelet counts, lower CD4 cell counts, higher serum cryptococcal antigen titer, and a higher rate of fungemia. (Table 2). All patients with HIV-infection were infected by members of *C. neoformans* species complex, whereas 8 HIV-uninfected individuals (17.4%) were infected by species of the *C. gattii* species complex. Following combination of the two groups, the majority of patients had a CSF opening pressure > 20 cm.H_2_O, CSF pleocytosis with a predominance of mononuclear cells, high CSF protein levels, and a low CSF: serum glucose ratio. In comparison with HIV-uninfected individuals, patients with HIV-infection were more likely to have a high opening CSF pressure, had lower cerebrospinal fluid (CSF) protein level, higher CSF to serum glucose ratio, and a high CSF cryptococcal antigen titer. Abnormal computerized tomography was found more commonly in HIV-uninfected individuals with a predominance of hydrocephalus.

Multivariate analysis revealed that factors associated with being infected with HIV in patients with cryptococcal meningitis included age < 45 years (OR 8.70, 95% CI 1.78–42.62), white blood cells of < 5000 cells/cu.mm. (OR 7.18, 95% CI 1.45–35.61), and the presence of fungemia (OR 5.86, 95% CI 1.17–42.62).

### 3.3. Induction Therapy and Outcomes

The mainstay of treatment was amphotericin B with or without fluconazole or flucytosine. There were differences in the regimen, as shown in Table 3. The dose of amphotericin B varied from 0.7 to 1.0 mg/kg/day.

**Figure 2:**
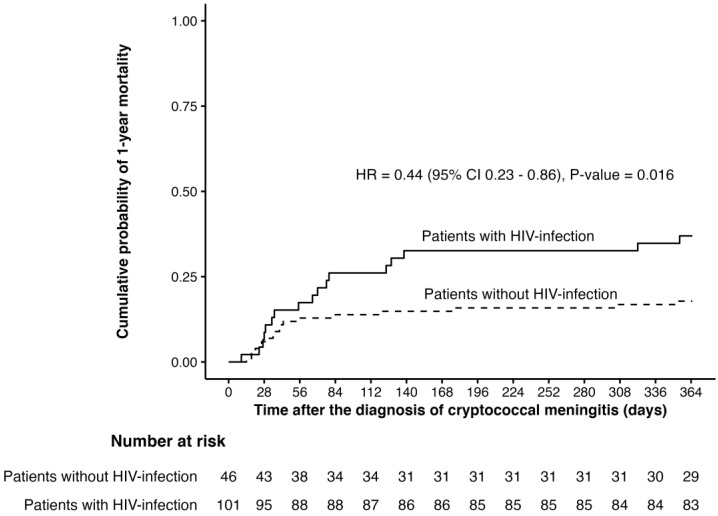
Cumulative probability of 1-year mortality among patients with cryptococcal meningitis by HIV-serostatus.

Overall, in-hospital, 30-day, 90-day, and 1-year all-cause mortality was 2% (3 patients), 7.5% (11 patients), 17.7% (26 patients), and 23.8% (35 patients), respectively. Patients with HIV-infection had a lower mortality rate in comparison to HIV-uninfected individuals. Median (IQR) time to death was 38 (25, 84) days and 66 (29, 124) days in patients with and without HIV-infection (*p*-value = 0.330). Patients who died were more likely to be older, were infected with HIV, experienced alteration of consciousness, and were infected by species of the *C. gattii* species complex (Figure 2). Multivariate analysis revealed factors associated with mortality were concurrent pneumocystis pneumonia (HR 5.44, 95% CI 1.55–19.15), alteration of consciousness (HR 2.94, 95% CI 1.42–6.10), infection caused by species of the *C. gattii* species complex (HR 4.19, 95% CI 1.39–12.62), and presence of anemia (HR 3.17, 95% CI 1.17–8.59) (Table 4). The length of hospital stay was longer for HIV-uninfected individuals.

## 4. Discussion

Cryptococcal meningitis is the most common cause of opportunistic adult meningitis, particularly in areas with a high prevalence of HIV-infection, especially in Southeast Asian countries [11]. The incidence of /666 meningitis declined dramatically after the increasing availability of antiretroviral therapy [12,13,14]. However, HIV-uninfected individuals are experiencing an increase in cryptococcal meningitis due to advances in medical technology, i.e., an increase in the rate of organ/stem cell transplants or an increase in availability of immunosuppressive drugs [15]. The current study found that 54.4% of HIV-uninfected individuals reported no underlying disease, which was comparable to the previous studies, which found a range of 55–67% [16,17,18,19]. Genetic factors might be a potential risk for immunocompetent HIV-uninfected patients [20]. Recent studies indicated that those of Chinese descent were more vulnerable to cryptococcal meningitis than other ethnicities [21,22,23]. In a 1970s study, 96% of HIV-uninfected Chinese Singaporeans diagnosed with cryptococcal meningitis were apparently healthy. Data from Hong Kong, Taiwan, and Shanghai also showed that most of the HIV-uninfected cryptococcal meningitis patients had apparently normal immune systems [24,25]. In addition, a recent cohort study revealed a correlation between Toll-like receptor (TLR) genes and cryptococcal infection in the Chinese population [20]. In individuals not infected with HIV, eight TLR single nucleotide polymorphisms displayed substantial genetic vulnerability to cryptococcal infection, whereas two polymorphisms were related to disease severity. These mutations enhanced the reaction to exposure to cryptococcal glucuronoxylomannan, resulting in a decrease in fungal clearance and an elevation in inflammatory cytokines. This evidence may indicate that people with Chinese ancestry are more easily susceptible to cryptococcal meningitis even in the absence of the risk of underlying disease [20].

Data from an earlier study showed that 63 out of 111 excreta samples from pigeons were found to be positive for *Cryptococcus* spp. [26]. This finding led to the assumption that pigeons and other bird species might harbor *Cryptococcus* spp., and contact with them would be associated with infection. In addition, northern Thailand, an area largely covered by moist deciduous forests, is a potential harbor for species of the *C. gattii* species complex [27,28]. However, data regarding environmental exposure were missing in the current study. Patients infected with species of the *C. gattii* species complex were all HIV-uninfected individuals (eight patients), and 75% of those (six patients) were apparently healthy. *C. gattii* species complex comprises five species: VGI (*C. gattii*), VGII (*C. deuterogattii*), VGIII (*C. bacillisporus*), VGIV (*C. tetragattii*), and VCIV/VCIIIc (*C. decagattii*). It was shown that VGI, VGII, and VGIII infect immunocompetent people, but VGIV mostly affects immunocompromised individuals [29,30]. In Asia, including Thailand, 73% of all isolates are VGI, followed by VGII at 19%; these incidence findings were similar to those related to Australia and New Zealand [29,30]. In contrast, VGII and VGIII predominate in America, whilst VGIV does so in Africa [30].

Consistent with previous studies, patients with HIV-infection were younger than HIV-uninfected individuals [19,31]. Patients with cryptococcal meningitis do not always have the classic signs associated with meningitis, specifically fever, nuchal rigidity, and altered sensorium [31,32,33]. However, we observed that alteration of consciousness was less prevalent in patients with HIV-infection, which is concordant with previous reports [18,19]. Typically, the duration of symptoms from onset to presentation is subacute [1]. However, this current study revealed that HIV-uninfected individuals came to hospitals earlier than individuals described within the previous reports (2 weeks in the current study vs. 6–12 weeks in a previous study) [1].

Laboratory indices from CSF also differed between patients with and without HIV-infection. Patients with HIV-infection were more likely to have anemia and leukopenia at the time of diagnosis, had a higher opening CSF pressure, higher serum cryptococcal antigen titer, and were more likely to have fungemia than patients without HIV-infection. Anemia and leukopenia may indicate chronic illness due to HIV-infection [34]. Higher opening CSF pressure, higher serum cryptococcal antigen titer, and the presence of fungemia represent a higher fungal load and disseminated infection in patients with HIV-infection due to low CD4 cell count [35,36]. However, HIV-uninfected individuals appeared to have a higher CSF protein concentration and a lower CSF: serum sugar ratio, which may indicate a more robust inflammatory response in immunocompetent hosts [37,38]. In this study, all HIV-infected patients were infected with members of *C. neoformans* species complex, whereas 17.4% of HIV-uninfected individuals were infected with *C. gattii*. This supported previous reports that *C. gattii* infected HIV-uninfected individuals more frequently than patients with HIV-infection [30,31,39,40,41]. Brain imaging was frequently abnormal, especially in HIV-uninfected individuals. Hydrocephalus appeared to be the most frequent abnormality seen in HIV-uninfected individuals, which corresponded to the findings in previous studies [31,42,43].

Amphotericin B combined with flucytosine is the induction therapy of choice for patients with cryptococcal meningitis regardless of HIV-serostatus [44]. However, as flucytosine was unavailable in the hospital before the year 2020, fluconazole was mainly used in combination with amphotericin B during the study period.

Compared to the study carried out in 2005–2010, in-hospital, 90-day, and 1-year mortality rates in patients with HIV-infection were shown to have declined dramatically in the current study (1.0% vs. 24.1%, 13.9% vs. 32.4%, and 17.8% vs. 52.2%, respectively) [45]. Mortality rate among patients without HIV-infection was higher than HIV-infected patients and was driven by the 1-year mortality. This might be explained by the fact that patients with HIV-infection have immune restoration after initiation of antiretroviral therapy [46,47]. In contrary, some patients without HIV-infection in this study may receive long-term immunosuppressive agents and may suffer from their underlying diseases or other opportunistic infections contributing to death. However, HIV-serostatus was not found to be associated with mortality after adjusting for confounders. Factors associated with mortality from multivariate analysis included infections caused by the presence of alteration of consciousness, the concurrence of pneumocystis pneumonia, infection caused by species of the *C. gattii* species complex, and anemia. The presence of alteration of consciousness may indicate a high opening CSF pressure due to a high fungal burden [16,31,48]. Concurrent pneumocystis pneumonia may reflect the low CD4 cell count and may put patients at risk of other opportunistic infections during a 1-year period and may lead to higher mortality. An association between cryptococcal meningitis caused by species of the *C. gattii* species complex and high mortality in comparison to members of *C. neoformans* species complex were reported [30,48,49,50,51,52]. As also observed in this study, infection caused by species of the *C. gattii* species complex occurred in HIV-uninfected individuals and may lead to a delay in diagnosis and treatment. In addition, species of the *C. gattii* species complex may not respond as well to amphotericin B in comparison to members of *C. neoformans* species complex [53]. Hypoalbuminemia and older age were reported in association with mortality in other studies [54,55].

There were several limitations in this study. First, due to the nature of all retrospective studies, some data may be missing and lead to misinterpretation of the results. Second, due to the small sample size, if other factors associated with mortality existed, some correlations may not have been captured.

## 5. Conclusions

The incidence of cryptococcal meningitis in patients with and without HIV-infection differed in some aspects. Patients infected with HIV were more likely to be younger, have leukopenia, and fungemia. An awareness of these differences may help to improve patient care for people with cryptococcal meningitis.

## Figures and Tables

**Table 1:** Characteristics of cryptococcal meningitis between patients with and without HIV-infection.

Characteristics	HIV-Infected Patients (n = 101)	HIV-Uninfected Patients (n = 46)	*p*-Value
Male—n (%)	66 (65.4)	29 (63.0)	0.787
Age at diagnosis (years)—mean (SD)	45.0 (12.4)	61.9 (16.3)	<0.001
Opportunistic infections—n (%)	19 (18.8)	3 (6.5)	0.053
▪ Candidiasis	7 (6.9)	0 (0)	0.099
▪ Tuberculosis	6 (5.9)	2 (4.3)	1.000
▪ *Pneumocystis jiroveci* pneumonia	4 (4.0)	1 (2.2)	1.000
▪ Cytomegalovirus infection	4 (4.0)	0 (0)	0.310
▪ Herpes simplex virus infection	4 (4.0)	0 (0)	0.310
▪ Progressive multifocal leukoencephalopathy	0 (0)	1 (2.2)	0.313
Co-infections—n (%)			
Hepatitis B virus infection	6 (5.9)	0 (0)	0.177
Hepatitis C virus infection	0 (0)	1 (2.2)	0.313
Syphilis	1 (1.0)	0 (0)	1.000
Charlson Comorbidity Index—median (IQR)	6 (6, 7)	3 (2, 4)	<0.001
Duration of symptoms before diagnosis (days)—median (IQR)	14 (4, 30)	14 (7, 30)	0.921
Presenting symptoms—n (%)			
▪ Fever	64 (63.4)	27 (58.7)	0.589
▪ Headache	73 (72.3)	29 (63.0)	0.260
▪ Alteration of consciousness	14 (13.9)	18 (39.1)	0.001
▪ Nausea/vomiting	29 (28.7)	5 (10.9)	0.017
▪ Seizure	15 (14.9)	7 (15.2)	0.954
▪ Diplopia	14 (13.9)	6 (13.0)	0.893
▪ Gait disturbance	1 (1.0)	3 (6.5)	0.091
▪ Speech problems	2 (2.0)	2 (4.3)	0.589
▪ Weakness	9 (8.9)	3 (6.5)	0.754
▪ Sensory deficit	2 (2.0)	3 (6.5)	0.177

**Table 2:** Laboratory findings pertinent to cryptococcal meningitis in patients with and without HIV-infection.

Characteristics	HIV-Infected Patients (n = 101)	HIV-Uninfected Patients (n = 46)	*p*-Value
**Blood tests**			
Hemoglobin—g/dL—mean (SD)	10.9 (2.2)	11.8 (1.9)	0.027
Hemoglobin ≤ 10 g/dL	39 (84.8)	64 (63.4)	0.009
White blood cells—cells/cu.mm.—median (IQR)	5100 (3800–6900)	8985 (6290–14830)	<0.001
White blood cells >15,000—cells/cu.mm.—n (%)	2 (2.0)	11 (23.9)	<0.001
White blood cells <5000—cells/cu.mm.—n (%)	49 (48.5)	6 (13.0)	<0.001
Platelets ×1000/cu.mm—mean (SD)	240.3 (120.2)	309.6 (138.1)	0.002
CD4 cell count—median (IQR)	23 (10, 68) (n = 96)	226 (150, 449) (n = 6)	<0.001
Serum cryptococcal antigen titer—n (%)			<0.001
Undetectable	0/97 (0.0)	3/40 (7.5)	
1:10	9/97 (9.3)	4/40 (10.0)	
1:100	17/97 (17.5)	19/40 (47.5)	
1:1000	32/97 (33.0)	9/40 (22.5)	
1:10,000	38/97 (39.2)	5/40 (12.50)	
>1:10,000	1/97 (1.0)	0/40 (0.00)	
Serum cryptococcal antigen titer ≥1:10,000—n (%)	39/97 (40.2)	5/40 (12.5)	0.002
Hemoculture grew *Cryptococcus* species—n (%)	44 (43.6)	10 (21.7)	0.011
**Cerebrospinal fluid (CSF) analysis**			
Opening pressure > 20 cm.H_2_O- n (%)	69 (72.6)	17 (51.5)	0.026
White blood cells –cells/cu.mm.—median (IQR)	49 (10, 220)	90 (20, 183)	0.130
Mononuclear—%—median (IQR)	100.0 (86.0, 100.0)	100.0 (66.0, 100.0)	0.481
Protein—g/dL—median (IQR)	65.5 (45.0, 108.5)	117.5 (71.0, 224.0)	<0.001
CSF: serum sugar ratio –%	36.5 (16.3)	24.9 (16.3)	<0.001
India ink positive—n (%)	66/100 (66.0)	23/44 (52.3)	0.118
Culture grew *Cryptococcus* species—n (%)	75/100 (75.0)	30/44 (68.2)	0.396
▪ *Cryptococcus neoformans* species complex	101 (100.0)	38 (82.6)	<0.001
▪ *Cryptococcus gattii* species complex	0 (0.0)	8 (17.4)	<0.001
CSF cryptococcal antigen titer—n (%)			0.212
Undetectable	4/99 (4.0)	0 (0.0)	
1:10	9/99 (9.1)	8/44 (18.2)	
1:100	26/99 (26.3)	14/44 (31.8)	
1:1000	30/99 (30.3)	14/44 (31.8)	
1:10,000	29/99 (29.3)	7/44 (15.9)	
>1:10,000	1/99 (1.0)	1/44 (2.3)	
CSF cryptococcal antigen titer ≥1:10,000—n (%)	30/99 (30.3)	8/44 (18.2)	0.130
**Imaging study**—n (%)			
Chest radiograph abnormality	27 (26.7)	16 (34.8)	0.320
Computerized tomography abnormality	61 (60.4)	37 (80.4)	0.017
▪ Hypodensity lesion	15 (14.9)	10 (21.7)	0.303
▪ Gelatinous pseudocyst	10 (9.9)	5 (10.9)	1.000
▪ Hydrocephalus	16 (15.8)	24 (52.2)	<0.001
▪ Abscess	1 (1.0)	2 (4.3)	0.230
▪ Leptomeningeal enhancement	20 (19.8)	10 (21.7)	0.787
▪ Infarction	11 (10.9)	3 (6.5)	0.550

**Table 3:** Treatment and outcomes of cryptococcal meningitis between patients with and without HIV-infection.

Characteristics	HIV-Infected Patients (n = 101)	HIV-Uninfected Patients (n = 46)	*p*-Value
**Induction therapy—n (%)**			0.001
Amphotericin B+ flucytosine	0 (0.0)	5 (10.9)	
Amphotericin B + fluconazole	65 (64.4)	35 (76.1)	
Amphotericin + flucytosine + fluconazole	1 (1.0)	0 (0.0)	
Amphotericin B	30 (29.7)	6 (13.0)	
Fluconazole	5 (5.0)	0 (0.0)	
Dose –mg/kg/day—n (%)			0.183
0.7	40 (41.7)	15 (32.6)	
0.8	0 (0.0)	1 (2.2)	
1.0	56 (58.3)	30 (65.2)	
Duration of induction—days—median (IQR)	14 (13, 16)	14 (13, 27)	0.088
**Mortality—n (%)**	18 (17.8)	17 (37.0)	0.020
Median time to death—days—median (IQR)	38 (25, 84) (n = 18)	66 (29, 124) (n = 17)	0.030
In-hospital—n (%)	1 (1.0)	2 (4.3)	0.231
30-day—n (%)	6 (5.9)	5 (10.9)	0.320
90-day—n (%)	14 (13.9)	12 (26.1)	0.101
1-year—n (%)	18 (17.8)	17 (37.0)	0.020
**Other outcomes**			
Amphotericin-induced acute kidney injury—n (%)	24 (23.8)	16 (34.8)	0.169
Length of hospital stay—days—median (IQR)	15 (8, 23)	19 (14, 36)	0.002

**Table 4:** Characteristics of cryptococcal meningitis between patients who survived and patients who died.

Characteristics	Patients Who Survived (n = 112)	Patients Who Died (n = 35)	*p*-Value
Male—n (%)	73 (65.2)	22 (62.9)	0.841
Age at diagnosis (years)—mean (SD)	48.9 (15.3)	54.8 (16.6)	0.045
HIV infection	83 (74.1)	18 (51.4)	0.020
Opportunistic infections—n (%)	15 (13.4)	7 (20)	0.415
▪ Candidiasis	5 (4.5)	2 (5.7)	0.671
▪ Tuberculosis	6 (5.4)	2 (5.7)	1.000
▪ *Pneumocystis jiroveci* pneumonia	2 (1.8)	3 (8.6)	0.088
▪ Cytomegalovirus infection	3 (2.7)	1 (2.9)	1.000
▪ Herpes simplex virus infection	3 (2.7)	1 (2.9)	1.000
▪ Progressive multifocal leukoencephalopathy	1 (2.2)	0 (0)	1.000
Charlson Comorbidity Index—median (IQR)	6 (6, 7)	6 (3, 7)	0.182
Median (IQR) duration of symptoms before diagnosis (days)	14 (5.5, 30)	14 (3, 30)	0.411
Presenting symptoms—n (%)			
▪ Fever	69 (61.6)	22 (62.9)	0.894
▪ Alteration of consciousness	16 (14.3)	16 (45.7)	<0.001
▪ Seizure	14 (12.5)	8 (22.9)	0.134
**Laboratory findings**			
Hemoglobin ≤ 10 g/dL	73 (65.2)	30 (85.7)	0.021
White blood cell count >20,000 cells/cu.mm.—n (%)	4 (3.6)	4 (11.4)	0.092
Platelets ×1000/cu.mm—mean (SD)	260.1 (128.8)	267.9 (134.2)	0.757
CD4 cell count—median (IQR)	26.5 (11, 78)	31 (11, 159.5)	0.519
Serum cryptococcal antigen titer >=1:1000—n (%)	66 (62.3)	19 (61.3)	0.992
Hemoculture grew *Cryptococcus* species—n (%)	43 (38.4)	11 (31.4)	0.456
**Cerebrospinal fluid (CSF) analysis**			
Opening pressure > 20 cm.H_2_O- n (%)	69 (61.6)	17 (48.6)	0.826
White blood cells –cells/cu.mm.—median (IQR)	66 (10, 229)	50 (10, 147)	0.323
Protein—g/dL—median (IQR)	77.5 (50, 125.5)	95.5 (50, 194)	0.375
CSF: serum sugar ratio –%	32.8 (15.9)	33.1 (21.1)	0.950
Culture grew *Cryptococcus* species—n (%)	82 (73.2)	23 (65.7)	0.880
▪ *Cryptococcus neoformans* species complex	110 (98.2)	29 (82.9)	0.002
▪ *Cryptococcus gattii species complex*	2 (1.8)	6 (17.1)	
CSF cryptococcal antigen titer ≥1:1000—n (%)	62 (55.9)	20 (62.5)	0.503
**Induction therapy—n (%)**			0.039
Amphotericin B combination with fluconazole or flucytosine	75 (67.0)	31 (88.6)	
Amphotericin B monotherapy	32 (28.6)	4 (11.4)	
Fluconazole	5 (4.5)	0 (0)	

## Data Availability

Some of the data will be shared upon request.
